# Analyzing the influence of oblique incidence on quantitative backscattering tissue polarimetry: a pilot *ex vivo* study

**DOI:** 10.1117/1.JBO.28.10.102905

**Published:** 2023-08-07

**Authors:** Zheng Zhang, Conghui Shao, Honghui He, Chao He, Shaoxiong Liu, Hui Ma

**Affiliations:** a Tsinghua University, Institute of Biopharmaceutical and Health Engineering, Tsinghua Shenzhen International Graduate School, Guangdong Research Center of Polarization Imaging and Measurement Engineering Technology, Shenzhen Key Laboratory for Minimal Invasive Medical Technologies, Shenzhen, China; b Tsinghua University, Department of Physics, Beijing, China; c University of Oxford, Department of Engineering Science, Oxford, United Kingdom; d Shenzhen Sixth People’s Hospital (Nanshan Hospital), Huazhong University of Science and Technology, Union Shenzhen Hospital, Shenzhen, China

**Keywords:** polarimetry, Mueller matrix, scattering imaging, endoscopy, Monte Carlo simulation

## Abstract

**Significance:**

Among the available polarimetric techniques, backscattering Mueller matrix (MM) polarimetry provides a promising non-contact and quantitative tool for *in vivo* tissue detection and clinical diagnosis. To eliminate the surface reflection from the sample cost-effectively, the non-collinear backscattering MM imaging setup always has an oblique incidence. Meanwhile, for practical organ cavities imaged using polarimetric gastrointestinal endoscopy, the uneven tissue surfaces can induce various relative oblique incidences inevitably, which can affect the polarimetry in a complicated manner and needs to be considered for detailed study.

**Aim:**

The purpose of this study is to systematically analyze the influence of oblique incidence on backscattering tissue polarimetry.

**Approach:**

We measured the MMs of experimental phantom and *ex vivo* tissues with different incident angles and adopted a Monte Carlo simulation program based on cylindrical scattering model for further verification and analysis. Meanwhile, the results were quantitatively evaluated using the Fourier transform, basic statistics, and frequency distribution histograms.

**Results:**

Oblique incidence can induce different changes on non-periodic, two-periodic, and four-periodic MM elements, leading to false-positive and false-negative polarization information for tissue polarimetry. Moreover, a prominent oblique incidence can bring more dramatic signal variations, such as phase retardance and element transposition.

**Conclusions:**

The findings presented in this study give some crucial criterions of appropriate incident angle selections for *in vivo* polarimetric endoscopy and other applications and can also be valuable references for studying how to minimize the influence further.

## 1 Introduction

Polarimetry is attracting increasing attention nowadays, due to its unique ability to obtain microstructural and optical information of tissue samples label-free.^1^^–^^4^ Among the available polarimetric techniques, Mueller matrix (MM) imaging can comprehensively characterize the polarization-related properties of media, including diattenuation, retardance, and depolarization, which are of great importance in biomedical studies and clinical trials.^5^^–^^7^ Recently, transmission MM microscopy has shown potential in detection of various abnormal and pathological tissues, such as breast cancer,^8^ cervical cancer,^9^ colon cancer,^10^ skin cancer,^11^^,^^12^ liver cirrhosis,^13^^,^^14^ gastrointestinal cancer,^15^^,^^16^ thyroid cancer,^17^ and lung cancer.^18^ Meanwhile, the backscattering MM polarimetry provides a promising non-contact and quantitative tool for *ex-vivo* measurement of bulk tissue samples,^6^^,^^7^^,^^19^^–^^22^ *in-vivo* imaging of skin tissue,^23^^–^^26^ and endoscopic detection of internal organs.^27^^–^^31^

Specifically, for the measurement of bulk tissue specimens, a backscattering MM polarimetry setup whose polarization state generator (PSG) and polarization state analyzer (PSA) are located at the same side of the sample is necessary. Currently, there are two backscattering polarimetry schemes, namely the collinear reflection mode with normal incidence^32^ and the non-collinear reflection one with obliquely incident illumination.^5^^,^^19^^–^^26^^,^^33^ For MM imaging, ideal collinear reflection equipment can provide accurate measurement results, avoiding the influence from the spatial orientation of the sample. However, in practice, the collinear reflection mode brings other side effects, including direct back-reflection signals from the sample and intrinsic residual polarization effect induced from the polarization components, which require more efforts and cost to deal with.^34^ In contrast, the non-collinear reflection MM equipment has the ability to eliminate the surface reflection from the sample cost-effectively using an oblique incidence. Thus, it has been increasingly used in biomedical studies and applications.^5^ On the other hand, for practical organ cavities imaged using polarimetric gastrointestinal endoscopy, the uneven tissue surfaces can induce various relative oblique incidences inevitably, which needs to be considered for detailed study.

Some recent studies have noted that the obliquely incident illumination can affect the MM measurement in a complicated manner,^35^^,^^36^ which hinders the effectiveness for distinguishing different sources of anisotropies. For instance, a Monte Carlo simulation based on the sphere–cylinder scattering model showed that the residue rotation dependence of MM increases with the oblique incidence angle.^37^ Thus, for real applications, a systematic analysis of the influence of oblique incidence on backscattering MM polarimetry is needed.

In this study, we measure the MMs of an anisotropic tissue phantom, a porcine liver tissue, and a human gastric muscularis tissue sample under different obliquely incident illuminations. The *ex vivo* experimental results demonstrate that oblique incidence of backscattering polarimetry can induce different changes on non-periodic, two-periodic, and four-periodic MM elements, which are comprehensively evaluated using the Fourier transform, basic statistics, and frequency distribution histograms (FDHs). Moreover, we adopt a Monte Carlo simulation program based on cylindrical scattering model for further analysis. Both the experiments and simulations confirm that a prominent oblique incidence can bring more dramatic signal variations, such as phase retardance and element transposition to MM. The findings presented in this study give some crucial confidence boundaries of appropriate incident angle selections for MM imaging based on specific properties extraction. It also indicates that some important structural information of the tissue sample may be obtained through the symmetry breaking and period degeneracy of MM elements, as the incident angle modulated.

## 2 Methods and Materials

### 2.1 Sample and Experimental Setup

**Fig. 1:**
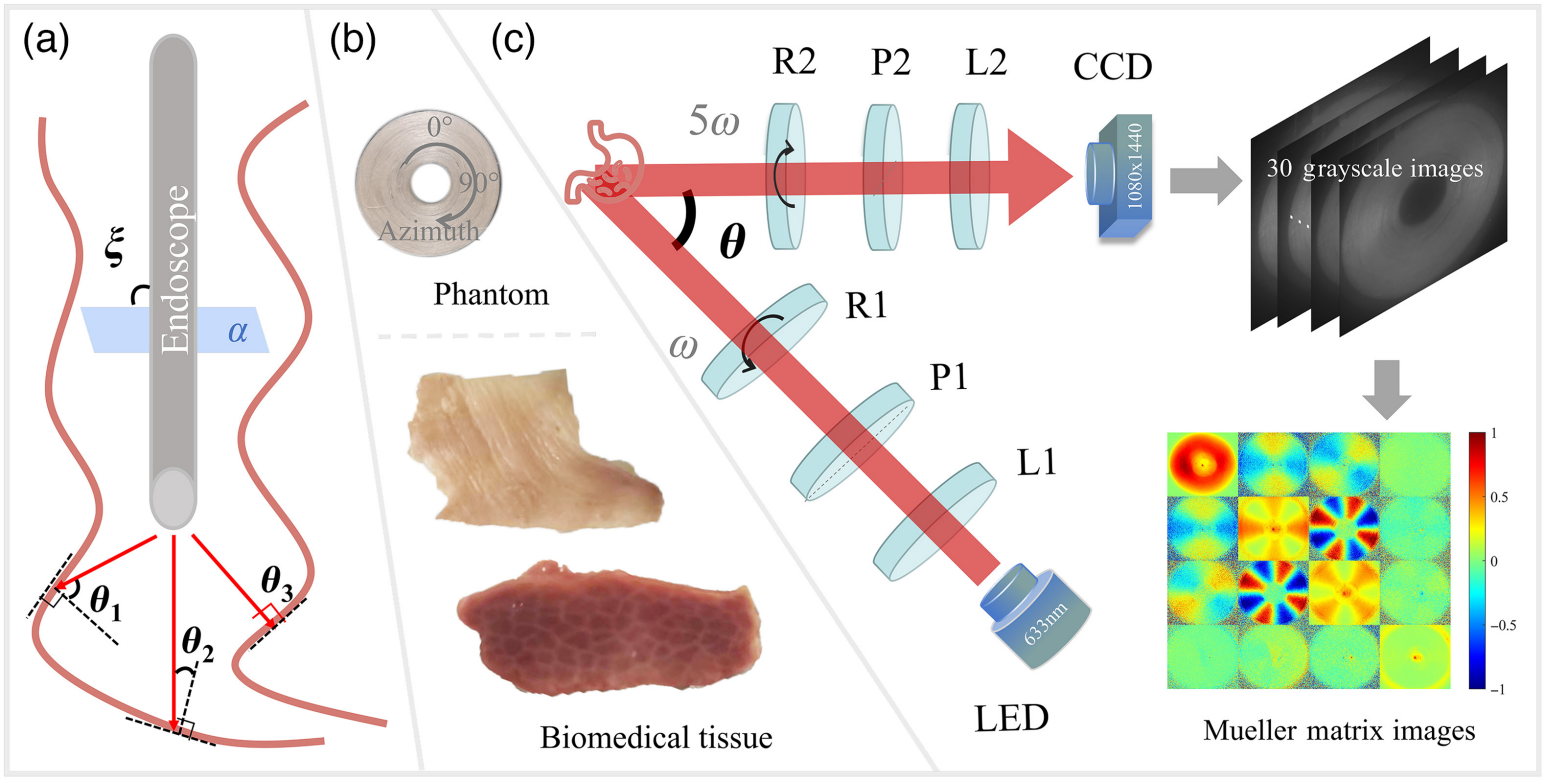
Schematics of setups and samples: (a) oblique incidence induced by tissue surface in gastrointestinal endoscopy; (b) concentrically aligned silk phantom, porcine liver, and human gastric muscularis tissues; and (c) backscattering DRR experimental setup and flowchart of MM imaging acquisition. L1 and L2, lenses; P1 and P2, polarizers; R1 and R2, rotating quarter-wave plates; θ, the oblique incident illumination angle. During each measurement, R1 and R2 rotate with constant rates (ω for R1 and 5ω for R2).

For practical *in vivo* tissue polarimetry, such as polarization gastrointestinal endoscopy, even though it is at a normal incidence ξ with the incident plane α shown in Fig. 1(a), a variety of relatively oblique incident angles exist according to the uneven tissue interfaces at different locations. For instance, $\theta_{1}$, $\theta_{2}$, and $\theta_{3}$ are prominent oblique incidence, moderate oblique incidence, and normal incidence, respectively. Such a kind of oblique incidence induced by tissue sample’s surface is inevitably and brings influence on polarization measurement quantitatively.

To investigate its influence, as shown in Fig. 1(b), we designed an anisotropic phantom consisted of concentrically aligned silk fibers, adopted a porcine liver tissue sample and a human gastric muscularis tissue sample for *ex vivo* polarimetric imaging. The human gastric muscularis tissue was prepared and provided by Shenzhen Sixth People’s Hospital (Nanshan Hospital) Huazhong University of Science and Technology Union Shenzhen Hospital. This work was approved by the Ethics Committee of Tsinghua Shenzhen International Graduate School, Tsinghua University. In particular, the phantom generated the MMs of cylindrical scatterers along all directions in the imaging X−Y plane in single measurement,^35^^,^^36^^,^^38^ whereas the porcine liver tissue was used for distinctive observation of depolarization and retardance, and the human gastric muscularis sample was analyzed for diattenuation and total anisotropy.

The experimental setup used in this study is a typical backscattering MM polarimetric system based on a dual-rotating retarder (DRR) scheme.^39^^,^^40^ As shown in Fig. 1(c), the obliquely incident illumination light from an LED (3 W, 633 nm, Δλ=20  nm, Daheng Optic, Beijing, China) passes through the PSG, which consists of an objective lens L1 (Hengyang Optic, Guangzhou, China) to produce parallel beam, a fixed polarizer P1, and a rotatable quarter-wave plate R1 to generate light with different polarization states. Then, the polarized light scattered by the sample transmits through the PSA, which consists of a rotatable quarter-wave plate R2, a fixed linear polarizer P2, and a lens L2 (Hengyang Optic, Guangzhou, China), correspondingly. Both the polarizers P1 and P2 (extinction ratio > 1000:1, Thorlabs, New Jersey, United States) are fixed in horizontal direction, and the rotatable quarter-wave plates R1 and R2 (Daheng Optic, Beijing, China) are controlled by the servo motor drivers (PRM1Z8, Thorlabs, New Jersey, United States) with a fixed angular rotating rate for 30 times (ω for R1 and 5ω for R2). Then, the photons are received by the CMOS camera (MV-CA016-10UM, 1440×1080  pixels, 12-bit, Hikvision, China) to calculate the MM elements of the sample, and the single frame acquisition time is <0.8  s. Here, all the intensity matrix elements extracted from the 30 grayscale images are related to the I as inputs. Considering the repetition of polarization states during each acquisition cycle, we rewrite the coefficient of angular rotating rate to 2nωt, and the Fourier coefficients $\alpha_{n}$ and $\beta_{n}$ can be calculated as^41^

$$I=\alpha_{0}+\overset{12}{\underset{n=1}{\sum }}(\alpha_{n}\;\cos\;n\omega t+\beta_{n}\;\sin\;n\omega t).$$ (1)

Before measurement, the backscattering MM polarimetric setup was calibrated using air as the standard sample to make sure that the system error is within 1%. More details of the calibration procedure can be found in Ref. 42. Here, by varying the angle θ between the PSG and PSA arms, all the MMs of the sample are obtained as the raw data for further analysis of the relationship between oblique incident illuminations and the elements.

### 2.2 Analysis Methods

For quantitative analysis, a ring area with radius of 200 pixels of each MM element is taken by the interpolation method to calculate its averaged value and aims to construct the azimuthal dependent curves of the MM elements from 0 deg to 360 deg. Then, some basic statistics, such as the mean and kurtosis, are used to evaluate the influence of incidence on the azimuthal dependent curves of MM elements. In details, the mean reveals the variation in the distribution of the overall value for element pixels, whereas the kurtosis reveals how outlier-prone a distribution is. Specifically, a normal distribution has a kurtosis value of 3, and the peak with larger kurtosis preferentially represents the whole azimuthal dependent curves for multi-peaks. The statistics used in this study are given as μ and k as follows:

$$\mu =\frac{1}{n}\sum_{i=1}^{n}p_{i},$$ (2)

$$k=\frac{\frac{1}{n}\overset{n}{\underset{i=1}{\sum }}{\left(p_{i}-\mu \right)}^{4}}{{\left(\frac{1}{n}\overset{n}{\underset{i=1}{\sum }}{\left(p_{i}-\mu \right)}^{2}\right)}^{2}},$$ (3)

where $p_{i}$ is the pixel value and n is the number of interpolated pixels for azimuthal dependent curves.

Our previous studies have demonstrated that the azimuthal dependent curves of the MM elements can be fitted to some trigonometric functions with different periods.^5^^,^^35^ To quantitatively evaluate the variations of the curve’s intensity and period under oblique incidences, we extracted the curve’s peak and valley values marked as P and V. Then, we calculated the differences between adjacent peak–peak and peak–valley values marked as P−P and P−V values, respectively. Meanwhile, the distance between the twin peaks is recorded as the P−P gap, which can reveal the period degeneracy of azimuthal dependent curve.

For the quantitative analysis of *ex vivo* tissues, we calculate the light intensity distributions for the sample to transform the 2D images into 1D FDHs. The horizontal shift of the FDH curves can reflect the overall variations (decrease or increase) of the MM elements’ values, whereas the shape can reflect the distribution changes of the MM elements, which can be used to evaluate the influence of oblique incidence.

Since the periodic signal can be treated as a sum of the baseline and a series of harmonics, here, we adopt the fast Fourier transform (FFT) method to analyze such periodic variation. After FFT, the amplitudes of each harmonic component are given as $A_{n}$ in Eq. (4), which can be calculated according to Eq. (5) related to Eq. (1)

$$x(t)=\alpha_{0}+\overset{180}{\underset{n=1}{\sum }}A_{n}\;\cos(n\omega_{0}t+\varphi_{n}),$$ (4)

$$A_{n}=\sqrt{\alpha_{n}^{2}+\beta_{n}^{2}},$$ (5)

where $\alpha_{0}$ is the baseline called direct-current component as well, $n\omega_{0}$ is the harmonic frequency of n components. The proportion of different harmonic components reflects the periodic variations of the MM elements comprehensively. It should also be noted that taking the FFT for an asymmetric periodic square wave with amplitude A, as $\alpha_{n}=0$, the amplitudes of each harmonic component can be calculated as

$$A_{n}=\sqrt{\alpha_{n}+\beta_{n}}=|\beta_{n}|=\frac{2}{T}\int_{-\frac{T}{2}}^{\frac{T}{2}}A\;\sin\;n\omega_{0}tdt=\frac{4}{T}\int_{0}^{\frac{T}{2}}A\;\sin\;n\omega_{0}tdt=\frac{2A}{n\pi }[1-\cos\;n\pi ],\;=\{\begin{array}{cc} \frac{4A}{n\pi }, & n=1,3,5,\ldots ,\\ 0, & n=2,4,6,\ldots . \end{array}$$ (6)

Thus, square wave signal has more odd harmonic components than even harmonic components, especially with a certain coefficient ratio for ideal results in Eq. (7). Then, based on three main harmonic components, we defined a coefficient $C_{s}$ to describe this square wave characteristic in Eq. (8), which can be used to verify the period degeneracy in Secs. 3.2–3.4 when $C_{s}$ is close to 0

$$A_{1}=3\;A_{3}=5\;A_{5}=7\;A_{7}=nA_{n},\;n=1,3,5,\ldots ,$$ (7)

$$C_{S}=|3A_{3}-A_{1}|+|5A_{5}-A_{1}|+|7A_{7}-A_{1}|.$$ (8)

### 2.3 Monte Carlo Simulation

For further study and verification, we also adopt a Monte Carlo simulation based on the cylinder scattering model, in which the concentrically aligned silks are simplified as infinitely long cylindrical scatterers along different directions. In the simulation, trajectories and polarization states of scattered photons are tracked synchronously as they propagate in the medium. Detailed settings of simulation parameters have been presented in our previous study.^35^

## 3 Results and Discussions

### 3.1 Azimuthal Dependent Curves of MM Elements Under Different Oblique Incidences

**Fig. 2:**
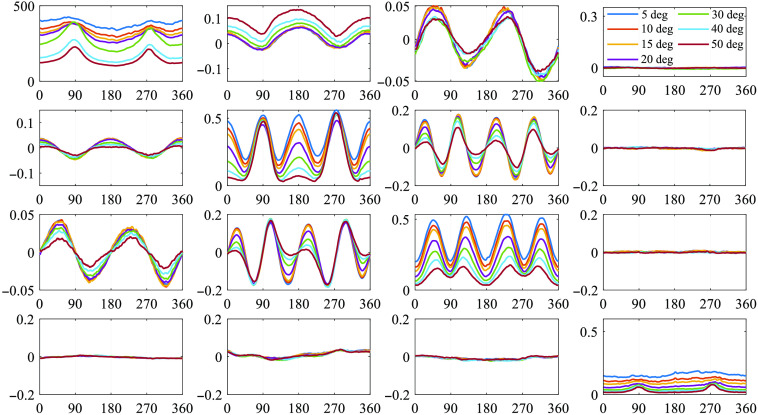
Azimuthal dependent curves of 4×4 MM elements under oblique incidences of 5 deg, 10 deg, 15 deg, 20 deg, 30 deg, 40 deg, and 50 deg, respectively. The horizontal axis shows the azimuth angle of the silk fibers, the vertical axis is the value of MM elements. All the elements are normalized by M11.

The azimuthal dependent curves of full 4×4 MM elements under different oblique incidences between 5 deg and 50 deg are shown in Fig. 2. It can be observed that different oblique incidences bring specific changes on different elements, which can be divided into three categories: (1) the non-periodic diagonal elements M11 and M44, whose variations can be quantified using mean and kurtosis values; (2) the two-periodic off-diagonal elements M12, M21, M13, and M31, which are related to linear diattenuation and polarizance properties of the medium, representing symmetry breaking; and (3) the four-periodic central-block elements M22, M33, M23, and M32, which are related to linear depolarization and anisotropy of the medium, representing period degeneracy. Hence, in the following sections, we will discuss the detailed influence of oblique incidence on the three groups of MM elements.

**Fig. 3:**
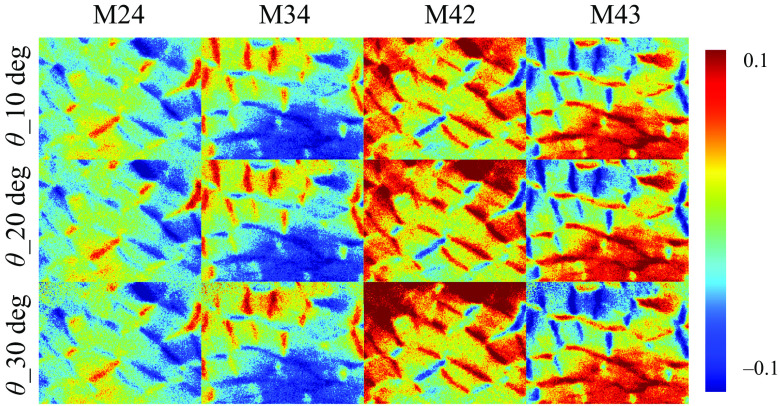
M24, M34, M42, and M43 images of porcine liver tissue under obliquely incident illuminations of 10 deg, 20 deg, and 30 deg, respectively.

It also should be noted from Fig. 2 that the M14, M24, M34, M41, M42, and M43 elements do not change significantly under different obliquely incident illuminations. These elements mainly reflect the retardance, circular diattenuation, and polarizance properties, which are limited in backscattering imaging results of silk phantom.^35^^,^^36^^,^^38^ In fact, for backscattering tissue polarimetry, the 3×3 MM is often adopted to provide main structural features of bulk tissues conveniently.^43^ For a demonstration, Fig. 3 shows the robustness of the corresponding elements for characterizing the retardance property of the porcine liver tissue sample under different oblique incidences. It can be seen that the changes of the M24, M34, M42, and M43 elements are limited as the incident angle θ increases within 20 deg to 30 deg.

### 3.2 Variation of the Mean and Kurtosis in Non-Periodic Diagonal Elements

**Fig. 4:**
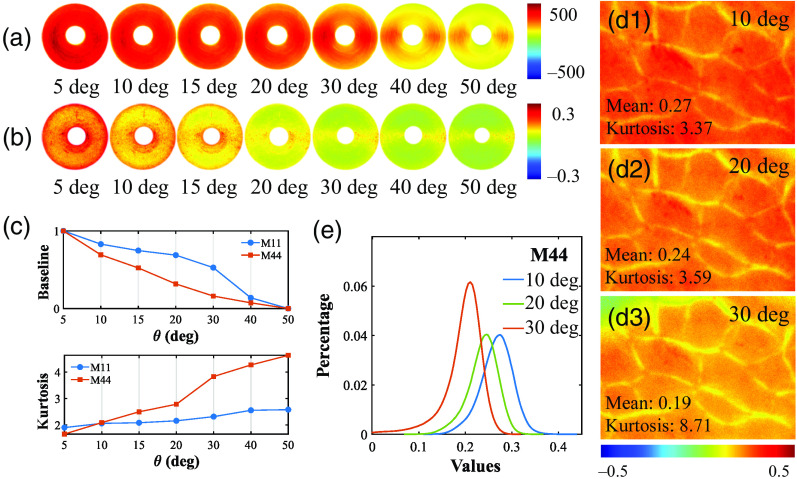
MM images and analysis of concentrically aligned silk phantom and porcine liver tissue sample under different obliquely incident illuminations: (a) M11 of the silk phantom; (b) M44 of the silk phantom; (c) mean and kurtosis of the silk phantom. (d1) M44 of porcine liver tissue at 10 deg incidence; (d2) M44 of porcine liver tissue at 20 deg incidence; (d3) M44 of porcine liver tissue at 30 deg incidence. (e) FDH for the M44 of porcine liver tissue.

The diagonal elements M11 and M44 reflect the unpolarized backscattering intensity and circular depolarization, respectively.^44^^,^^45^ Ideally, the M11 and M44 elements intensities should be azimuthal independent.^35^^,^^46^^,^^47^ However, from Figs. 4(a) and 4(b), it can be noticed that as θ increased, the intensity values of M11 and M44 decrease, and the silk fibers along vertical direction (90 deg and 270 deg) show stronger signals compared to those along the horizontal direction, which can also be observed from Fig. 2. To quantify such intensity changes, we calculated the mean and kurtosis of the M11 and M44 curves according to Eqs. (2) and (3) shown in Fig. 4(c). Specifically, when θ becomes larger than 20 deg, the kurtosis of M44 increases sharply from 2.78 to 3.84, which suggests that θ within 20 deg would be considered preferably to extract reliable circular depolarization information from the M44.

For an *ex vivo* tissue verification, we calculated the basic statistics of M44 images of porcine liver tissue sample with θ increasing from 10 deg to 30 deg, as shown in Figs. 4(d1)–4(d3), which reveals the enhancement and annihilation of the photons scattered from the tissue at specific azimuths. The finding is also supported by the leftward shift of the M44 intensity distribution curve of porcine liver tissue shown in Fig. 4(e). In summary, we should be aware of and minimize such false-positive circular depolarization induced by incidence to avoid inaccurate conclusions in tissue polarimetry, especially when θ becomes larger than 20 deg.

### 3.3 Symmetry Breaking in Two-Periodic Off-Diagonal Elements

**Fig. 5:**
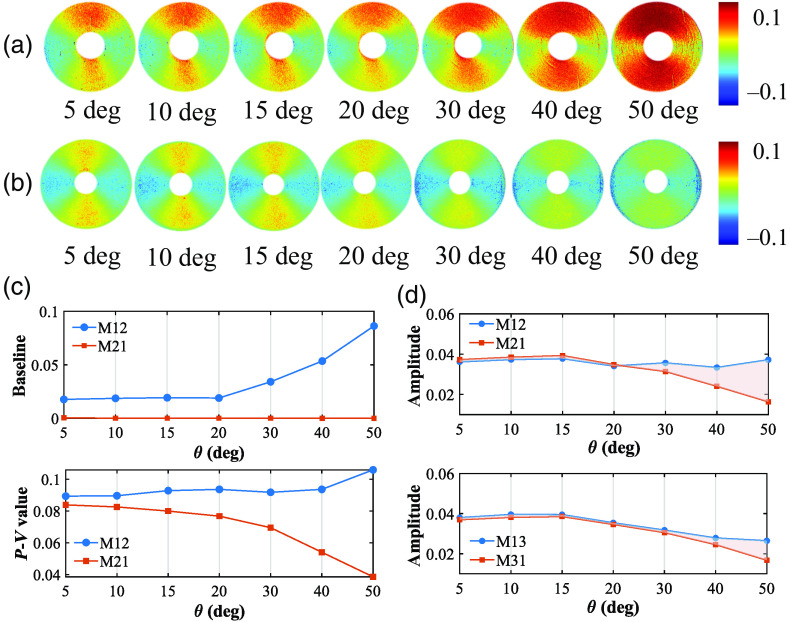
MM images and analysis of concentrically aligned silk phantom under different obliquely incident illuminations: (a) M12; (b) M21; (c) baseline and peak-to-valley value, calculated by the M12 and M21 in 1 period between 0 deg and 180 deg; and (d) amplitude of the third harmonic component after FFT, calculated by the M12 and M21, M13, and M31, respectively.

The symmetric properties between different elements tell us abundant optical and structural information of the sample.^18^ Ideally, the azimuthal dependent curves of M12 and M21, M13 and M31, which represent the linear diattenuation and polarizance properties of the silks, should be perfect trigonometric functions with two periods.^5^ Moreover, the M12 and M21 pairs, together with the M13 and M31 pairs, should both be symmetric.^35^ However, Fig. 2 shows that such kind of symmetry can be broken. We can see it more clearly from the MM images of concentrically aligned silk in Figs. 5(a) and 5(b). The opposite trend of both the baseline and the P−V value in Fig. 5(c) verifies such a symmetry breaking, as well.

Moreover, we took FFT to analyze the baseline to the eighth harmonic component in sequence of these two-periodic off-diagonal elements. Among the harmonic components, the third harmonic accounts for the largest proportion, which were separately shown in Fig. 5(d) to confirm such a symmetry breaking. In addition, compared to the M13 and M31 pair shown in Fig. 5(d) (bottom), the symmetry breaking between the M12 and M21 pair presented in Fig. 5(d) (top) is much more significant after 20 deg incidence, whereas the M12 grow abnormally large at special azimuths. It may be relevant to the obliquely incident illumination scheme. For instance, an oblique incident illumination (oblique PSG arm) generates influence particularly on the linearly diattenuation elements, whereas an oblique analyzing arm (oblique PSA arm) generates influence particularly on linearly polarizance elements.

**Fig. 6:**
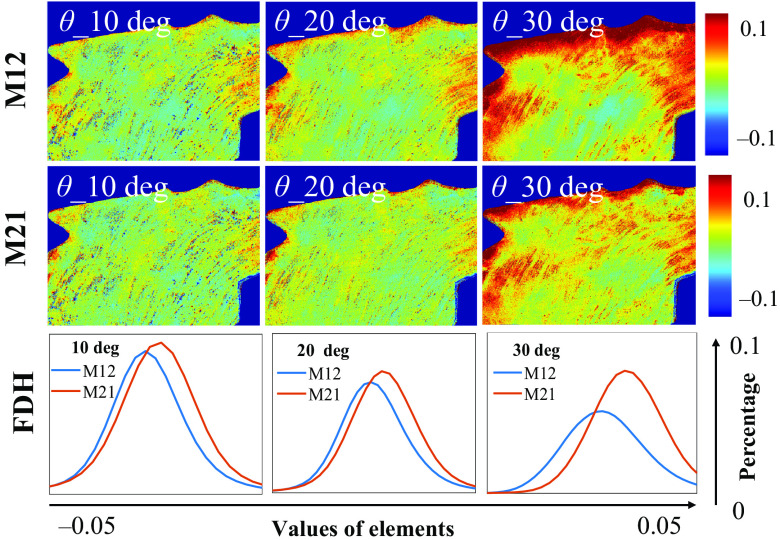
M12 and M21 images, and the corresponding FDH curves of human gastric muscularis tissue sample under obliquely incident angles at 10 deg, 20 deg, and 30 deg.

For an *ex vivo* tissue verification, we measured the M12 and M21 elements of human gastric muscularis tissue sample, as shown in Fig. 6. It can be observed that as θ increases, the elements’ values of specific areas change prominently, which can induce false-positive linear diattenuation. Meanwhile, Fig. 6 also indicates that the shapes between the FDHs of M12 and M21 varying from each other with θ increasing. In summary, an incident angle within 20 deg can be considered preferably for oblique incidence, set to extract reliable linear diattenuation.

### 3.4 Period Degeneracy in Four-Periodic Central-Block Elements

**Fig. 7:**
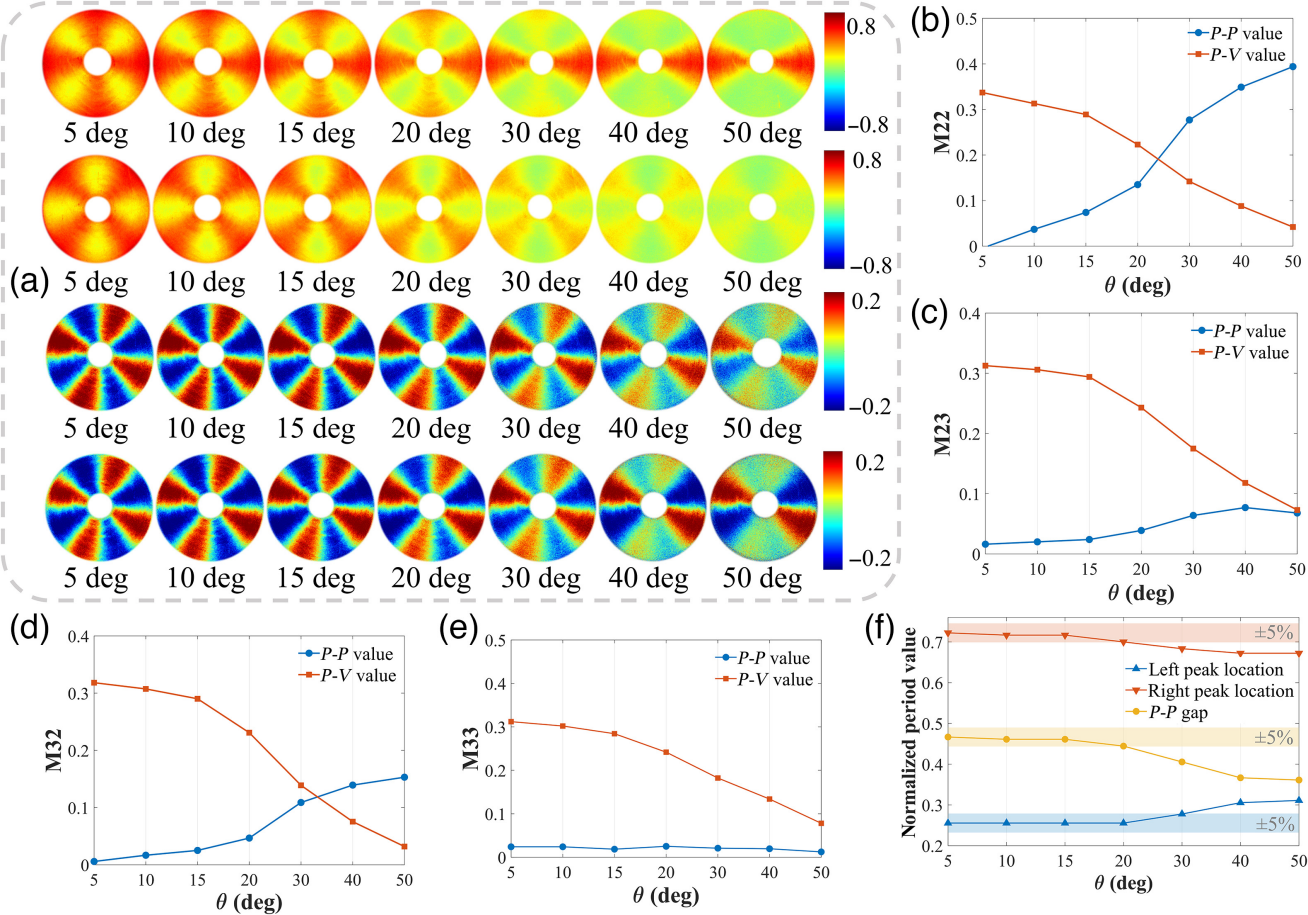
MM images and analysis of concentrically aligned silk phantom under different obliquely incident illuminations. (a) Images of M22, M33, M23, and M32 from above to below, respectively. (b)–(e) Comparison of P−P value represented by blue dots and P−V value represented by red squares of M22, M23, M32, and M33. The peak and valley values are taken from the first 2 periods of 0 deg to 180 deg. (f) Relative locations and gap between peak and valley in the 0 deg to 180 deg period of M33. The period length has been normalized.

The central-block MM elements M22, M33, M23, and M32, which are related to linear depolarization and the overall linear anisotropy properties, ideally should have trigonometric functions with four periods under normal incidence.^5^^,^^35^ However, Fig. 2 indicates that as θ increased, the four periods degenerated. Figure 7(a) further visualizes the period degeneracy of the central-block elements, gradually from four to two periods with different features.

For a more detailed observation of the diagonal M22 and M33 elements in the first 2 periods from 0 deg to 180 deg, we can see from Fig. 2 that as θ increases: (1) the M22, which is related to the 0 deg and 90 deg linear polarization components of both input and output Stokes vectors, has a sharp single-peak collapse at the special azimuth locations of 0 deg and 180 deg and (2) the M33, which is related to the 45 deg and 135 deg linear polarization components of both input and output Stokes vectors, has a sharp double-peak collapse at the special azimuth locations of 45 deg and 135 deg. Such different forms of period degeneracy make the curves of M22 and M33 under larger oblique incidences tend to be more similar as impulse and square wave signals, respectively. Hence, we list all the amplitudes of each harmonic component for M33 after FFT in Table 1. It can be seen that the odd harmonic components account more proportion than the even harmonic components, and the coefficient of square wave signal $C_{s}$ tends to 0 as θ increases, which is precisely the characteristics of square wave signal in Eq. (8).

**Table 1:** Amplitude of harmonic components of M33 under different oblique incidences. Bold values are intended to make odd harmonic components more observable.

θ (deg)	1st	2nd	3rd	4th	5th	6th	7th	8th	$C_{s}$
5	**0.361**	0.013	**0.016**	0.023	**0.149**	0.020	**0.011**	0.004	**0.981**
10	**0.320**	0.008	**0.020**	0.023	**0.148**	0.019	**0.012**	0.005	**0.916**
15	**0.292**	0.007	**0.025**	0.022	**0.143**	0.018	**0.012**	0.004	**0.848**
20	**0.237**	0.005	**0.032**	0.023	**0.120**	0.022	**0.012**	0.004	**0.657**
30	**0.184**	0.006	**0.039**	0.016	**0.092**	0.017	**0.015**	0.003	**0.422**
40	**0.143**	0.006	**0.043**	0.012	**0.066**	0.014	**0.015**	0.004	**0.239**
50	**0.106**	0.006	**0.041**	0.007	**0.041**	0.007	**0.012**	0.003	**0.138**

For further demonstrations of the period degeneracy, Figs. 7(b)–7(e) show the changes of P−P (blue dots) and P−V (red squares) values for the M22, M23, M32, and M33 elements. The opposite changing trends confirm the existences of period degeneracy. It is worth mentioning that, as θ increases, the characteristic of double-peak collapse for the M33 period degeneracy makes its P−P value vary little, as shown in Fig. 5(e), but the locations of the twin peaks get closer and closer. Figure 5(f) illustrates such kind of peaks convergence, whereas the P−P gap (yellow dots) is the distance between twin peaks accordingly. Obviously, the P−P gap gradually becomes smaller as the incident angle becomes larger than 15 deg. Thus, the results shown in Table 1 and Fig. 5 indicate that an incident angle within 15 deg should be considered preferably, set to extract reliable linear depolarization and anisotropy information from the central-block MM elements.

**Fig. 8:**
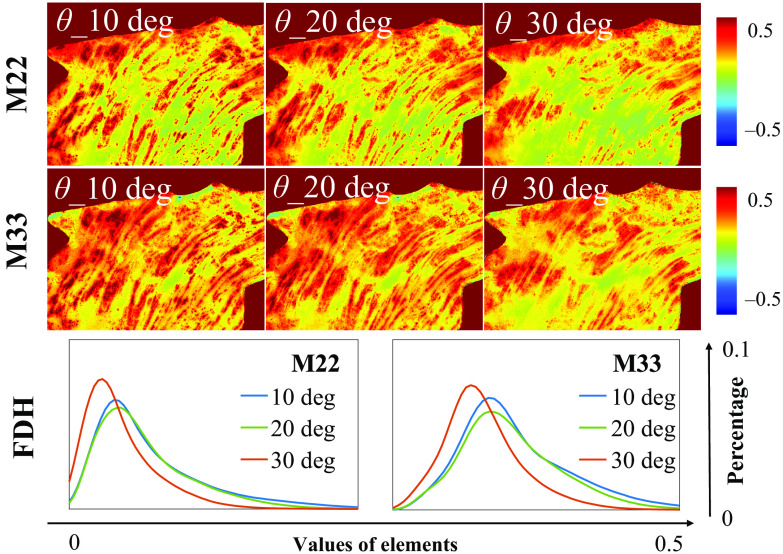
M22 and M33 images, and the corresponding FDH curves of human gastric muscularis tissue sample under obliquely incident angles at 10 deg, 20 deg, and 30 deg.

Moreover, such a kind of periodic degeneracy may lead to an annihilation of polarization information at particular azimuths, such as −45  deg to 45 deg, which can result in false-negative linear anisotropy in tissue polarimetry when the incident angle increased beyond a confidence boundary. For an *ex vivo* tissue demonstration, we observed the M22 and M33 of human gastric muscularis tissue sample experimentally shown in Fig. 8, from which the decrease of some pixel values can be seen in both the images and FDHs.

### 3.5 Dramatic Curve Variations with Prominent Oblique Incidence

**Fig. 9:**
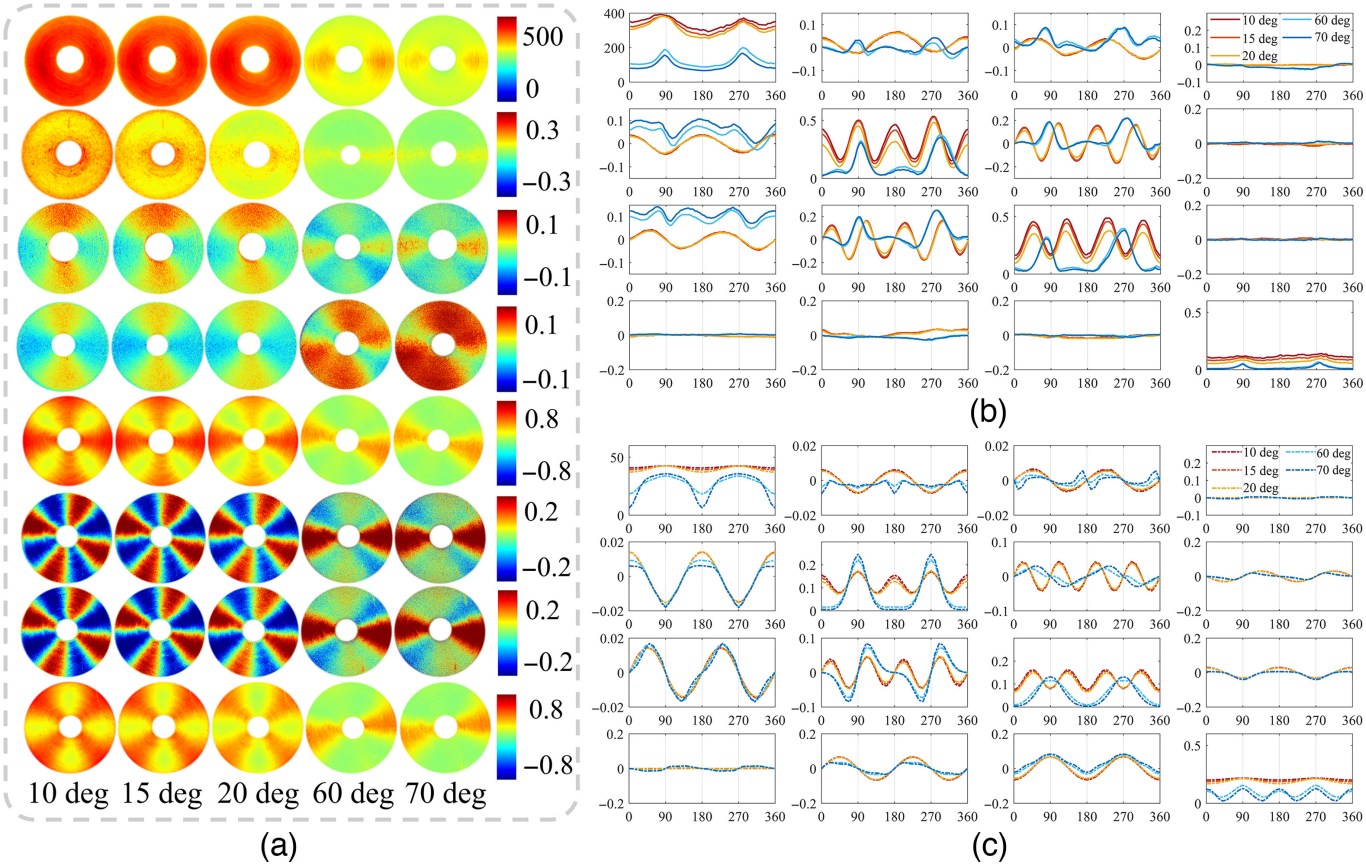
MM images and analysis of concentrically aligned silk phantom under different obliquely incident illuminations. (a) Images of the M11, M44, M12, M21, M22, M23, M32, and M33 under oblique incidences of 10 deg, 15 deg, 20 deg, 60 deg, and 70 deg from above to below, respectively. (b) and (c) Experimental and Monte Carlo simulated azimuthal dependent curves of 4×4 MM elements under oblique incidences of 10 deg, 15 deg, 20 deg, 60 deg, and 70 deg, respectively. All the elements are normalized by M11.

As θ increased beyond 50 deg, dramatic variations can happen for all the MM elements. It can be observed from Figs. 9(a) and 9(b) that there are great discrepancies of the element characteristics between prominent oblique incidence and moderate oblique incidence. In details, besides the various effects mentioned above, we can see that: (1) the two-periodic elements additionally transposed their values and (2) the baseline of the four-periodic elements decreases additionally accompanied by a certain phase retardance. For example, Fig. 9(c) shows the Monte Carlo simulated curves, which are consistent with the experimental results. Moreover, when the incident angle is modulated, some important structural information of the sample may be obtained through the characteristics of MM elements, such as symmetry breaking and period degeneracy.

## 4 Conclusions

In this study, we analyzed the influence of oblique incidence on quantitative backscattering tissue polarimetry. The different changes induced by oblique incidence on MM elements include: (1) variation of the mean and kurtosis in non-periodic diagonal elements M11 and M44; (2) symmetry breaking of the two-periodic off-diagonal elements M12, M21, M13, and M31; and (3) period degeneracy of the four-periodic central-block elements M22, M33, M23, and M32. Both *ex vivo* experimental images and quantitative analysis revealed the variation of depolarization information, false-positive linear diattenuation, and false-negative anisotropy resulted from incidence. It also indicated that some important structural information of the sample may be obtained through the symmetry breaking and period degeneracy of MM elements, as the incident angle modulated prominently. Moreover, the phase retardance and element transposition induced by an oblique incidence larger than 50 deg deserve to be noticed and carefully avoided. The findings presented in this study give some crucial criterions of appropriate incident angle selections for *in vivo* polarimetric endoscopy and other applications, and can be valuable references for studying how to minimize the influence further.

## Code, Data, and Materials Availability

Data underlying the results presented in this paper are not publicly available at this time but may be obtained from the authors upon reasonable request.
